# Specific manifestations of sandwich generation effect in deaf parents and CODA families

**DOI:** 10.1093/jdsade/enaf020

**Published:** 2025-05-14

**Authors:** Vít Dočekal, Eva Klimentová

**Affiliations:** Palacký University Olomouc, Faculty of Arts, Department of Sociology, Andragogy and Cultural Anthropology, Olomouc, Czech Republic; Palacký University Olomouc, Faculty of Arts, Department of Sociology, Andragogy and Cultural Anthropology, Olomouc, Czech Republic

## Abstract

The main group of interest in this study are deaf parents of hearing children and the aim is to describe the phenomenon of the inverted sandwich generation effect with deaf parents of hearing children. The basic research framework for this study was Interpretative Phenomenological Analysis as defined by Smith et al. (Smith, J. A., Larkin, M., & Flowers, P. (2009). Interpretative phenomenological analysis: Theory, method and research. Sage.). Five themes were defined—grandparent help, child help with interpretation and life support, help from neighbors and others, parental dependency, child independence, and interpretation of childhood and parenthood. These themes were interpreted by analyzing data from two groups of respondents—deaf parents and their hearing children (children of deaf adults). The main finding relates to the reverse sandwich model operating within these generations.

## Background

### The concept of “sandwich generation”

The sandwich generation is defined by Miller (1981, p. 419) as individuals “ready for relaxation and self-indulgence, only to find that their grown children are not quite independent and their parents have moved from autonomy to a degree of dependence.” A more accurate definition of the traditional sandwich generation is offered by Burke (2017, p. 4): “those sandwiched are in their 50s and 60s, with aging parents, children and perhaps grandchildren.” Other authors extend the range of years and argue that this is the middle generation of individuals between the ages of 35 and 64 (Synowiec-Pilat et al., 2022, p. 4). An entire book is devoted to the sandwich generation by Bertini (2011, p. 1), who defines it as “individuals who have come to midlife and find themselves ‘sandwiched’ between their children and their aging parents, nurturing, providing for, and filling what can seem like nonstop, too numerous, and perhaps even overwhelming needs of both their offspring and their elders.” Similarly, Parker and Patten (2013, p. 2) define the sandwich generation as “those who have a living parent age 65 or older and are either raising a child under age 18 or supporting a grown child.” These definitions, outlining the sandwich generation in relation to the surrounding generations for whom they provide help and support, are also useful for our investigation and understanding of some of the context. This specific generation is likely to expand its numbers in the coming years as baby boomers approach retirement. Additional reasons for this future growth are “delayed childbearing and increased female labor-force participation, especially of women, who are caught between the demands of child rearing and elder care while attempting to play a more demanding role in the work force” (Spillman & Pezzin, 2000, p. 347). This generation is also under pressure from above and below to experience “increased levels of stress and are exposed to high risk for mental strain” (Riley & Bowen, 2016, p. 57).

This appears to be a pivotal aspect. The manner of acting as pillars in the sandwich generation is related to the resolution of the conflict between the demands of the ascendant and descendant generations. This primarily concerns the financial and temporal resources that the sandwich generation devotes to caring for the two succeeding generations. While these two resources are limited and allocating them in one direction reduces the possibilities for caring for the second generation, emotional resources in this respect seem unlimited by external influences (Grundy & Henretta, 2006). However, perceiving this emotional capacity as unlimited can lead to negative consequences. A large number of studies report that a large proportion of caregivers in this family setting suffer from depression, anxiety, and other negative symptoms often associated with burnout syndrome. Sudarji et al. (2022) confirm the arise of emotional stress when both children and parents need attention. Their study aimed at women in sandwich generation mentions “feelings of sadness, guilt, and unstable emotions, and in terms of cognitive, forgetfulness, loss of concentration, and overthinking” (Sudarji et al., 2022, s. 363). However, it is not only emotional issues that arise for those in the sandwich generation. Research also shows negative physical effects on caregivers in this situation. (Tyas & Kusumaningrum, 2021, p. 834) report that “physical complaints include fatigue, severe migraine, nausea, dizziness, insomnia, knee and back pains, rheumatism, and frequent colds.” Noor and Isa (2020) who conducted research in Malaysia complement additional issues confronting the sandwich generation, such as financial restraints and savings issues, dim future, and lack of time (management).

On the other hand, two-sided care (to parents and children) can also have positive effects, as evidenced by Turgeman-Lupo et al. (2020), who report that one of the mechanisms that promote recovery is the happiness of children and their development. In contrast, Williams (2004) describes a positive effect heading in the opposite direction toward aging parents. Williams (2004) reports that “60% of those working and caring for an older person while still having children at home felt that caring for a senior was simply giving back what they had received, and 70% stated that the relationship was strengthened” (Williams, 2004, p. 9).

Given the unique circumstances of families with children of deaf adults (CODA), the intergenerational relationships within these families may display specific dynamics. These family structures often intersect with broader generational roles, such as caregiving and interdependence, which are commonly associated with the concept of the sandwich generation. Therefore, an exploration of how the sandwich generation effect manifests provides a valuable framework for CODA families’ situation. Not only Hortová and Souralová in the Czech context state that the effect of life expectancy increases the length of time that several generations live together, thus prolonging intergenerational assistance. Concurrently, the historical trend persists, wherein grandparents provided substantial financial assistance to the younger generation, including housing and childcare arrangements that enabled mothers to resume employment following parental leave (Hortová & Souralová, 2019). The described robust position of grandparents is also consistent with the findings of international surveys, wherein the most prevalent response under the item of contributions from individuals aged 55+ is the care of grandchildren. In a nationally representative survey, 82% of respondents indicated that they were contributing in this area, compared to 65% of the European Union average (Eurobarometer, 2012). Additionally, Albertini et al. present data on the sandwich effect in Eastern European countries, where citizens are less likely (compared to continental and northern European countries) to consider themselves socially sandwiched and to help their parents the least of all groups (Albertini et al., 2022). These results may indicate that the sandwich generation effect could be slightly reduced in local settings by involving grandparents in helping parents in general.

### CODA

The primary focus of this study is on deaf parents of hearing children. We perceive these individuals as a specific group with special needs. Their situation is different from deaf parents with deaf children in that CODA^1^ (see Bishop & Hicks, 2005, 2007, 2008; Preston, 1996; Singleton & Tittle, 2000; Filer & Filer, 2000) parents have to introduce their children to a hearing society, which is a different culture from the deaf culture. The distinction between the hearing and deaf cultures is elucidated by Padden and Humphries (2005, p. 3; 1988, p. 119), who emphasize that the discrepancy is not merely a dichotomy between the presence and absence of a sensory modality but rather a divergence at the level of language and the associated cultural elements. In addition to the differences in linguistic structure and form, the language used by the deaf community also differs from that used by the hearing population.

Not only Hortová and Souralová in the Czech context state that the effect of life expectancy increases the length of time that several generations live together, thus prolonging intergenerational assistance. Concurrently, the historical trend persists, wherein grandparents provided substantial financial assistance to the younger generation, including housing and childcare arrangements that enabled mothers to resume employment following parental leave (Hortová & Souralová, 2019). The described robust position of grandparents is also consistent with the findings of international surveys, wherein the most prevalent response under the item of contributions from individuals aged 55+ is the care of grandchildren. In a nationally representative survey, 82% of respondents indicated that they were contributing in this area, compared to 65% of the EU average (Eurobarometer, 2012). Additionally, Albertini et al. present data on the sandwich effect in Eastern European countries, where citizens are less likely (compared to continental and northern European countries) to consider themselves socially sandwiched and to help their parents the least of all groups (Albertini et al., 2022). These results may indicate that the sandwich generation effect could be slightly reduced in local settings by involving grandparents in helping parents in general.

## Methodology

This study was guided by the principles of Interpretative Phenomenological Analysis (IPA) as defined by Smith et al. (2009), emphasizing a focus on unique experiences and the idiographic approach to analysis. This approach aligns with the findings of Ladd (2003), who asserted that “the maximally useful way to research deaf culture at this point in time is to utilize bricolage, including methods which affirm introspection as a member of that culture.” In this research, we sought to investigate the parenting experiences of deaf parents and their hearing children (CODA), a group that exemplifies a unique context and dynamic. Given the hermeneutic dimension of IPA, our reflective experiences were also integrated into the analysis.

The study addressed three research questions. First, how did CODAs experience their childhood? Second, how do deaf parents of hearing children experience their parenting? Third, how do hearing children and their deaf parents interpret their relationships with other generations in the context of the so-called sandwich generation? These research questions evolved as the study progressed, guided by emerging themes and concepts, including the sandwich generation framework described by Miller (1981).

Data collection was carried out in two distinct phases, followed by one extra phase containing data analysis only. The first phase, conducted between 2014 and 2015, focused on CODA respondents and sought to address the first research question concerning their childhood experiences. The second phase, conducted from 2017 to 2019, centered on deaf parents of hearing children and aimed to explore their parenting experiences, corresponding to the second research question. The third phase emerged as a result of the data analysis from the previous two phases and aimed to answer the third research question, investigating intergenerational relationships and the implications of generational dependency. This question partially fulfils the characteristics of a so-called theory-driven research question or secondary research question (Smith et al., 2009), as we drew on an analogy already elaborated in the theory of the sandwich generation phenomenon.

Participants were selected through purposive sampling in collaboration with the Regional Union of the Deaf. The study included 14 CODAs aged between 18 and 41 years with prelingually deaf parents in the first phase, while the second phase involved seven prelingual deaf parents who had raised at least one hearing child over the age of 15. In this phase, the age of the parents was not recorded, as the primary criterion was the age of their child or children. To collect data, semistructured interviews were conducted and translated by a sign language interpreter for the deaf parent group. These interviews were audio-recorded, transcribed, and processed in MAXQDA software. Personal data were anonymized to protect the respondents, who were identified only as CODA or PAR to distinguish between children and parents.

In contextualizing the research, it is crucial to recognize the additional challenges faced by deaf parents globally and in the Czech Republic. During their adolescence, many were subjected to a segregated education system characterized by extensive auditory drills and lip-reading exercises rather than meaningful education. As Filippová and Hudáková (2016) describes, thinking was believed to be intrinsically linked to speech, which led to poor literacy and limited educational opportunities for deaf individuals. Kurková et al. (2018) confirmed the decline of “pure” sign language and the emphasis on oral education in special, segregated schools. This historical background led to entrenched barriers between sign and spoken language. Deaf parents often rely on their hearing children or other family members to bridge communication gaps, which places an unintended burden on CODAs. Heffernan and Nixon (2023) noted the protective role often assumed by CODAs, while other studies highlight the development of competencies such as empathy and problem-solving skills due to their bilingual and bicultural roles (Moroe & de Andrade, 2018a; Napier, 2021).

The data analysis process entailed several phases. Initially, a broad coding scheme was applied to the transcribed text. Examples of codes (out of a total of 111) include (grand) parents, bilingualism, “kids have to...,” child as mediator, tutoring, assessment by children, communication barrier, communication strategies in the family, deaf community, lack of communication, parents’ defense, children’s detachment, independence, shame, difficulties, interpreting children, cool, signing children, “we did it,” and others. Through iterative readings, the analysis became more focused, centering on the relationships between parents, children, and other generations. Descriptive, linguistic, and conceptual comments were incorporated to deepen the analysis. Graphical analysis tools in MAXQDA, such as Code Map and Wordcloud, provided additional perspectives on the data structure.

The researchers collaboratively defined the research questions, objectives, and strategy. Data collection was conducted by the co-author with the assistance of a sign language interpreter, while the lead author undertook data analysis through annotation, coding, and interpretation. Findings were developed through joint efforts, with reflective dialogues between researchers playing a crucial role. One researcher’s hard-of-hearing status and background in social work contributed to the interpretive process, while the other brought an external perspective without a personal connection to the topic. These reflections were incorporated into the final analysis to elucidate the relationship between the respondents’ and researchers’ interpretations.

To ensure transparency, it is important to clarify that data from earlier research on CODA childhood experiences served as the foundation for this study. The decision to revisit and reinterpret this data was driven by the emergence of generational dependency themes, prompting the exploration of relationships between CODAs, parents, and grandparents.

## Results

**Figure 1:**
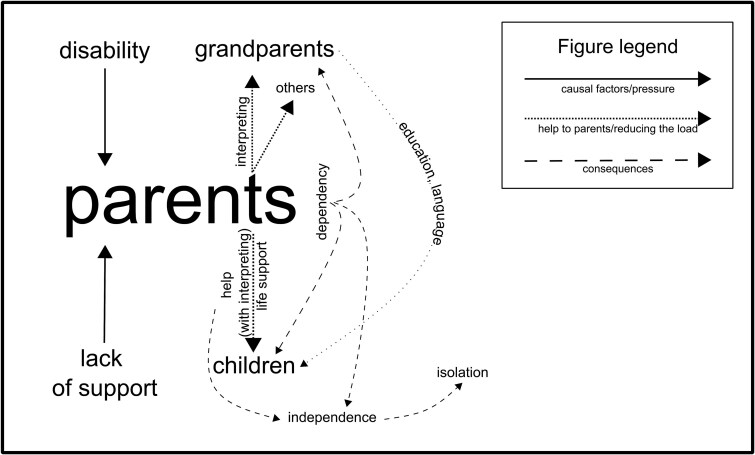
Research topics and relations.

This section will distinguish between three basic groups: children (whose quotes will be abbreviated as CODA), deaf parents (PAR), and grandparents (parents of deaf parents). These three groups form the basic structure of the sandwich generation. It should be noted that below, we separate only the CODA and parent (PAR) statements, not the statements of the individual respondents. We consider the interconnectedness of the individual statements, together with the isolated situations described mostly by the CODAs, to be a combination that jeopardizes the anonymity of our communication partners. The themes were created by clustering the individual codes into groups that made sense to the authors in the process of interpreting the content of the parts of the text marked by the individual codes and then naming them and looking for interconnections. These themes and the relationships between them are illustrated in Figure 1.

### Grandparent help

While it is not uncommon for grandparents to play a role in raising their grandchildren, experience of CODAs involved in our research shows a unique dynamic due to the communication barriers between their parents and the hearing world. This distinct situation is reflected in how grandparents often serve as both caregivers and facilitators of language. The narratives presented in this study illustrate the interconnectivity between the grandparents and the CODA children, with the grandparents playing a pivotal role in facilitating the children’s development. At the same time, they also ease the burden on the parents. “Grandma always helped a lot” (PAR)—several of these statements were made by parents in a similar way. Some CODAs spent a significant part of their childhood with grandparents “to learn how to talk because we wouldn’t have learned from our parents” (CODA). Coresidence with grandparents often provided CODAs in our research with a linguistic advantage. For example, one child recalled that although their father “tries to talk a lot,” the linguistic limitations posed by his deafness meant that “he doesn’t have that vocabulary” (CODA). Here, the grandparents’ role extended beyond mere homework assistance; it encompassed the transmission of hearing world language and culture, something their deaf parents could not fully provide. This highlights the dual role grandparents of our communication partners played as both educators and cultural mediators.

Additionally, assistance with academic tasks was provided: “my grandparents used to study with us, they taught us reading and math (...) and they used to write homework with us” (CODA). Assistance was also associated with the more challenging school curriculum: “And now in 8th and 9th grade it’s a little bit harder, I have no idea. So, the grandparents are always helping us, or they call and help” (PAR). This situation is associated with the lower expectations for deaf education in the national education system, which we previously described in one of our studies. “I didn’t even learn it and it is too hard. There used to be (...) more limited teaching for deaf people so we didn’t even learn it (PAR).” When asked who helped the children with school, one respondent replied, “always my Dad (note: grandfather” (PAR). In this case, the children’s grandfather was also heavily involved in their choice of secondary school.

In certain instances, however, the grandparents’ involvement extended beyond merely supporting the development of language competencies and assisting with school duties: “we were mostly brought up from a young age by my grandmother and grandfather and my parents were not involved in our upbringing, I think that even in terms of character I am very similar to my grandmother, I think I have nothing in common with either my mum or my dad and I think it is probably because we spent 90% of our time just with my grandparents; (...) it took a lot out of me, but I have to say that my grandmother made up for it in many ways” (CODA).

In addition to their role in the children’s development, grandparents acted as crucial intermediaries between the deaf parents and the hearing world. As one respondent noted: “as far as some dealings with the authorities and doctors, it was more my grandfather who went with our parents (CODA),” “as far as dealing with the authorities, it was my grandmother who dealt with our parents, it didn’t affect me as a child and even now they don’t need much” (CODA). This involvement went beyond family support; it positioned the grandparents as linguistic and social interpreters, bridging the gap between the parents and societal institutions. Such actions illustrate the broader societal role grandparents assumed in CODA families, where the ability to communicate in the hearing world became a critical asset. Notably, the assistance provided by the grandparents was largely voluntary, reflecting a desire to support rather than control their grandchildren’s upbringing. For instance, none of the respondents indicated any resentment or felt that their grandparents overstepped boundaries. This suggests a dynamic of mutual respect and collaboration, where grandparents were seen as allies in the children’s development, rather than authoritative figures.

### Child help with interpreting and life support

A central theme for many CODA children in our research involves their role as interpreters for their parents, particularly in navigating both routine and complex life situations. However, the extent of this involvement varies significantly depending on the family’s access to professional interpreting services and the willingness of the parents to involve their children in these tasks. Not only the children but also the parents confirmed that CODAs are used for routine and less common interpreting. This phenomenon is caused by the lack of support from the deaf community and the ease with which children enter or are included in interpreting. “The level of (note: professional) interpreters is really poor and when my mother goes to the doctor it is clear that she would rather have me there than a stranger” (CODA). Some CODAs also use interpreting to express a strong relationship with their parents: “I wouldn’t want a stranger to deal with these everyday situations and I want them to know that I am a support and that I will take care of them” (CODA). Conversely, even in formal settings, the institution was unaware of the availability of official interpreters or did not offer them: “I don’t remember anyone ever telling us that there were other options than us having to go everywhere” (CODA). This reflects a possible broader systemic failure in providing adequate support for deaf families, forcing children to assume responsibilities typically reserved for adults. The absence of formal guidance left many CODAs navigating complex bureaucratic systems on their own, which often resulted in long-term emotional impacts. This situation is not unique. In contrast, instances of guidance by authorities to parents regarding the use of interpreters are notably absent in the CODA interviews. As one parent recounted “the authorities told the parents that they would rather come with an interpreter, they themselves acknowledged there that it was probably beyond our capabilities” (CODA). This phenomenon is also evident in court hearings.

Interpreting became an integral part of many daily lives of CODAs in our research, extending even to routine tasks such as translating television programs. One child expressed frustration, stating, “it was really annoying how I had to interpret what they said on TV for them every day” (CODA). This highlights not only the sheer volume of interpreting responsibilities but also the emotional burden placed on young children, who may feel overwhelmed by the constant demand to serve as a language broker. Another parent noted: “my son sometimes helped us. I guess the hearing parents didn’t need that from the hearing children. But my son helped us a lot with some information” (PAR). This support that parents demand from their children also occurs, however, in more serious situations and starts at a young age. One interviewee stated, for example, that “we went to doctors and different institutions with them (...) from the second grade onwards” (CODA). Another communication partner stated that over time the responsibilities related to parental support began to shift from her grandparents to her: “when I was a little bit older and a little bit more capable, I started going to the office with them and everything fell on me” (CODA). The long-term impact of acting as interpreters from a young age left some CODAs feeling as though they were deprived of their childhood. One interviewee remarked, “I used to solve my parents’ problems as a kid, even though I didn’t understand them at all, and now I see that it took a piece of my childhood away from me.” This reveals the emotional cost of such responsibilities, as children were expected to manage adult issues, often without fully comprehending the situations they were navigating. The early assumption of such responsibilities can lead to premature maturity and the internalization of adult anxieties, impacting their development. The following stories present the unusual challenges faced by CODAs in early childhood. “At the age of six, I bought my parents a washing machine on hire purchase” (CODA). “My parents were waiting for me to grow up and be able to communicate, and only then did I go with my Dad maybe four times a month to submit and process housing applications (...) as a five-six-year-old boy, I arranged housing and it never surprised anyone” (CODA). “When Daddy was looking for a job, I went with him to all these different job interviews, so I have been through hundreds of interviews at my age” (CODA). “I was taken to all the meetings from as far as I can remember, including a court case where they were suing Daddy’s brother for my parents’ house and I was seven years old” (CODA). Almost fatefully, one respondent described the perceived life role of CODA: “we were born to help them” (CODA).

The reasons for this help can be found in the trust in their loved ones to convey information in a way that they understand. Thus, one respondent stated that the main reason CODAs served as primary interpreters was because parents did not trust or understand the other party when dealing with the situations mentioned above (CODA). “I handled it in a way that I also solved the merits of the matter, and most importantly I translated it into their language that they could understand” (CODA). “They are not equipped for life at all, for example, when an official letter comes, they don’t understand what they read anyway” (CODA). Support for parents persists into adulthood due to external demands, such as the need to comply with the expectations of their environment: “I’m going to the doctor with my Dad next week, (...) the doctor doesn’t want to deal with him himself, he told him to come with someone, he just wants him to have an interpreter with him” (CODA).

It is noteworthy that when assistance with interpreting from children or grandparents is unavailable, parents utilize the provision of professional services (which are provided at no cost). As one CODA participant observed, “when there was a point when none of us children were around and something had to be solved, there was this lady interpreter” (CODA). “My Dad is 76 now, so we don’t want to bother them any longer, because my Mum is sick, so we don’t want to bother them now, that’s why we come here (to draw on the Union’s interpreting services)” (PAR). “My daughter was older, so she actually handled everything (...), my son doesn’t want to make any calls. (...) And because my son didn’t want to, we started coming here, to the Regional Union of the Deaf, where we asked for a functional service (note: help with interpreting)” (PAR). Some parents are also aware of the burden that interpreting places on children. “I don’t want to force my children to interpret for me. I don’t. They themselves want to, or they don’t want to. It’s natural. I don’t like to push them to do it” (PAR) or another parent: “dealing with authorities (...) that’s for adults” (PAR). “When I go somewhere with my son, I don’t need him to interpret anything at all. When I go to the doctor, I ask for the interpreter” (PAR). While this perspective demonstrates an understanding of the potential stress, it also suggests that the responsibility often fell to the children by default, even if parents refrained from actively pushing them. This situation raises questions about whether CODAs truly had a choice in these situations, given the societal and familial pressures to assist.

### Help from the neighborhood and others

Other family members were also mentioned as a support group for parents, most often to explain school material to children: “my Dad has a cousin who (...) always came to help us” (CODA). However, the assistance of neighbors was far more prevalent. It is evident that in this region, deaf individuals do not mention friends, as these individuals are often also deaf and thus unable to provide assistance with spoken language or tutoring. As some of our respondents claim, neighbors often became the most accessible group for parents seeking assistance, particularly in the absence of professional interpreting services or family members with the necessary skills. However, the level of neighborly involvement varied across families, depending on the strength of social ties and the cultural context of the community. The assistance of neighbors enabled numerous children to become integrated into the hearing community of children and adults. As one parent noted, “the neighbors always took them to learn how to communicate with each other (...) or to hear words (...) the interception of words” (PAR). At the same time, thanks to neighbors, parents could check “if the children were speaking, articulating well (PAR),” and they also used the help to tutor their native language as a subject at school (PAR). Furthermore, establishing relationships offers the possibility of routine communication about parenting and child rearing: “I always ask my neighbor what to do (...), I always find out from others how they do it, if they give punishments or not. And then I know and I try to do it too” (PAR).

In addition, the school and teachers assist with school duties. It is common for students to receive tutoring in their native language and mathematics. This is often justified by the low level of mathematics in national schools for the deaf in the past. It is also evident that schools have a significant impact on children’s development of academic skills. One CODA respondent stated: “I didn’t learn to read until fifth grade when I met a fantastic teacher, and I owe it to her that I can read and that I have a secondary school and university degree” (CODA). School was also, however, a source of humiliation in the case of one respondent. “The teacher thought I was crazy” (PAR). This communication partner’s daughter was forced to sign and explain the family situation in front of the class and defend the parents’ competence. Other support groups were then classmates (tutoring) and speech therapists (language development).

### Parental dependency

Dependency appears in this chapter deliberately outside the framework of assistance from other groups, as we perceive it differently in our interpretation. While help is perceived here as a positive mechanism (if we leave aside some aspects such as the inadequate use of children as interpreters), the topic of dependency also leans toward a more negative interpretation of the condition. For the respondents in this study, parental dependence emerged as a significant theme, particularly in how they described their relationships with their deaf parents and grandparents. While these findings provide important insights into the lived experiences of CODAs, they (as well as other parts of the results) are specific to the communication partners in this research and may not fully represent the experiences of all CODA families. This is based on the aforementioned relationships with grandparents and children, which are based on helping in both simple and more complex situations. This dependency can also stem from the grandparents’ relationships with their parents. One CODA respondent shared that their grandmother reinforced the expectation of parental dependency: “My grandmother is supportive in my parental dependency and even keeps telling me: “take care of them, help them, they are your parents” (CODA). Another respondent confirmed this tendency: “All my life my grandmother was always telling my Mum that she is a poor person who needs to be helped and she acts like it” (CODA), or “my Dad has never been independent, he always needs help, he is so used to it and I think that it’s not normal, I don’t think anyone at home is like that” (CODA). In one family, the grandparents initially prevented the deaf mother from residing with her husband. “Grandpa was afraid that she couldn’t cook, she couldn’t take care of the child, she could only do laundry and clean, that’s it. And it just took years before she was allowed to move in with her own husband with whom she had a child” (CODA). These quotes highlight how intergenerational attitudes toward care can perpetuate cycles of dependency, as grandparents may view the role of children as caregivers, regardless of the parents’ capabilities. Such expectations, passed down through generations, can contribute to a sense of duty or obligation that CODA children may internalize over time.

Several CODA respondents expressed deep concerns about their parents’ vulnerability to manipulation or misunderstanding in formal settings. Our respondents shared various situations related to this topic. “Deaf people are very vulnerable victims (...) they are able to sign something and even something irreversible and maybe go into debt for years,” “I always felt like I had to watch them, even before the sales events” (CODA), “you have to be careful all the time, so that Mummy doesn’t sign something somewhere and get into trouble” (CODA), “Daddy has to commute sometimes and then I worry about him, he has to change trains and then he comes back at night and then my boyfriend and I really prefer going with him” (CODA). The independence of some parents also deteriorates rapidly with advancing age, as described in another testimony: “Daddy is unable to do anything anymore, not even go to the post office, to the bank, now lately he is not even able to write a text message by himself” (CODA). This highlights the significant emotional labor undertaken by CODA children, who often find themselves acting as both interpreters and protectors in various scenarios. The constant vigilance required to prevent harm underscores a dynamic where the child assumes a parental role, adding to the emotional toll CODA children often face. However, in the cases described by the respondents in this study, some parents demonstrated an increased level of independence after their children left home. For some, the absence of their children forced a degree of self-reliance, as illustrated by one respondent who shared the response of her daughter: “You do it yourself, you’re grown up, go, I’m not going to do anything, you do it yourself” (PAR). In some cases, respondents described their parents as almost independent of help. “My parents only have a problem when they need to phone somewhere” (CODA).

Some CODA communicators described a degree of parental helplessness to the point of viewing parents as their own children. This phenomenon is worthy of consideration in the context of this study, given the topic under investigation. The sandwich generation, which is typically burdened by the demands of caring for the adjacent generation, is relieved of at least one of these pressures in the case of our core group (that is, deaf parents). However, this relief is accompanied by the creation of additional pressures on the adjacent generation. “They’re not parents to me, they’re more like sort of my children, it’s probably silly to put it like that, but that’s the way it is” (CODA). Children of deaf adults in these situations mention that parents “don’t deal with things like we do all the time, they’re spared a lot of things, that’s a fact - they don’t even know about them” (CODA). A certain subtle concern for animation and spiritualization of parents’ lives also emerges: “I try to take my Dad to our house every weekend, we take him with my boyfriend somewhere for culture, to the zoo, to the cinema (...) he also loves to shop so much and so we take him to IKEA and there he walks around and looks at things and he’s so happy” (CODA).

Some children perceive parental dependence as a negative aspect of their upbringing. As one CODA respondent stated: “I don’t actually remember a time in my life when I had peace of mind (CODA),” “I think they take my whole life for granted that I help them, sometimes their helplessness interferes with my own life, I’m not able to manage everything” (CODA).

### Child independence

The aforementioned parental dependency has consequences for children—especially in terms of their own independence and overload. Based on the accounts of the respondents in this study, many CODAs reported not receiving strong parental support from childhood, which may have contributed to their development as independent and self-reliant individuals. However, this experience is not universal, and individual experiences of independence and parental support vary.

In the current study, several CODAs reported experiencing a strong sense of independence in school-related responsibilities, often relying on support from extended family or peers. However, this experience is not universal, and the role of external support systems varies between families. It is essential to recognize that the specific circumstances of these respondents may have influenced their particular experiences of independence. It appears that assistance with learning is beyond the capabilities of parents at a certain age due to the lower level of schooling previously designated for the deaf. One CODA stated, “we taught ourselves because my parents, not that they were less intelligent (...), but the education system back then, it was not that up to standard, in fact they didn’t give a damn back then, so they didn’t teach them much” (CODA). The impact of this insufficiency is reflected in the following statements: “...my parents never studied with us, they didn’t understand a lot of the stuff we were learning and they didn’t know what to help us with” (CODA), “I don’t remember my mother or anyone ever sitting down with us and asking what we needed to learn” (CODA), “my parents never read with me at all and I did my homework by myself, they just signed it but they didn’t even read it” (CODA). Some CODAs also perceive a lack of support for studying in general. “My parents never encouraged me to study and I didn’t want to do it myself, I did my homework by myself and I didn’t learn to read until I was at school” (CODA). Nevertheless, this is not the experience of all families. One mother, for instance, actively assisted her children with their schoolwork, stating “when homework was done or the children had to count something, we did it together, it was no problem” (PAR).

In certain instances, the situation reached a point where CODAs involved in this study were able to select their educational pathways independently, without parental input. “I didn’t consult it with anyone, I just wrote out the application form on my own, my parents just signed it (CODA, “I chose the school myself, I also handled the application form on my own, in fact everything I did from primary school onwards was just my own ambition” (CODA). Another CODA emphasized their independence in educational decisions: “I chose the school myself; I also handled the application form on my own” (CODA). These responses suggest that some CODAs may develop a high level of self-reliance at a young age, particularly when parents are not actively involved in educational decisions. However, this level of independence might be viewed as a coping mechanism developed in response to their unique family dynamics, rather than a typical developmental trajectory. Furthermore, parents corroborate the assertion that children are expected to be independent in school. As one parent noted: “the children had to learn on their own as well. My son has a lot of problems with mathematics, as far as languages are concerned, they have to learn by themselves because we are not able to help at all” (PAR).

Among the CODAs interviewed for this study, many reported a sense of independence and loneliness outside of school life. This reflects the unique dynamics observed in this sample but cannot be generalized to all CODAs. Parents discuss the development of their children’s specific character. “In her childhood, my daughter had to fight for everything, to make phone calls, to communicate with doctors, I guess somehow it sort of made her stronger” (PAR), “that’s why she’s used to making her own phone calls, to sorting things out (...) and she has such character, she is so strict” (PAR), “my daughter was really smart from a young age, she was used to (...), she had to try (...), she was independent and she got used to it afterwards” (PAR). CODAs also indicate the necessity for independence in becoming accustomed to this situation. “I was used to earn everything on my own because I knew that they (parents) wouldn’t give me anything” (CODA). In some cases, CODAs’ loneliness is supported by the general isolation of deaf parents from the hearing world and thus from their children.

Another reason for the children’s isolation could be the physical isolation during holidays, which, according to several respondents, looked as follows: “my parents used to go with their deaf friends, we don’t know what it is like to go on trips to the mountains with ours” (CODA), “they always preferred other deaf people, they went on trips with them and left us at home with Grandma” (CODA). This is not the rule, however, as one parent confirmed: “we all went on holiday together” (PAR) as well as one of the children: “we used to go on holiday with our parents and also to the seaside a lot (...) it was because there were always a few families of deaf parents who had hearing children and we would go” (CODA).

The physical separation of CODAs from their parents was not the sole factor contributing to their overall isolation. Psychological separation also played a role. As one CODA stated, “it completely took away that relationship with them, I just don’t come to my Mum to share something with her, I don’t do that because I haven’t developed that love for my parents” (CODA). Frequently, voices from CODA confirm the lack of warm affection from parents: “I love them more than they love me, I have a terrible problem and that is lack of love” (CODA), “I started dating boys quite early on, to get out of the house I guess, and I also needed that feeling of being loved a lot” (CODA), “I don’t have that kind of mother who advises her daughter when she is in trouble” (CODA). The absence of a family model as the pinnacle of children’s detachment from their parents is described by other CODAs: “they are not for family, not at all, which is so interesting” (CODA), “they never took us as their children, they never knew how to deal with us” (CODA). Some parents may impress upon their children that they are relieved that their children are now independent: “I can see in my parents how they are happy that they finally have their peace and don’t have to worry about their children any more” (CODA).

### Childhood and parenthood evaluation

The text primarily concerns itself with the elements that draw attention to the relief of the situation for deaf parents and the effects of this relief, particularly on their hearing children. Despite the impression that this text is predominantly critical of the fulfillment of the parental role by deaf parents, it is important to note that respondents in this study often evaluate their childhood and parenting as positive or normal, although this evaluation is undoubtedly specific. “When I look at other deaf and hearing children or even deaf children, I think the parenting is exactly the same, except for the school” (CODA). One respondent shared a positive experience of parental support, despite the parents’ deafness: “I used to take them to these concerts and... it seemed like they were just normal parents” (CODA). This reflects an interesting dynamic where the respondent finds validation in parental presence rather than their ability to actively engage in the event itself. The fact that the parents attended, even though they couldn’t hear, suggests that for this respondent, emotional presence and perceived normalcy were more important than practical functionality. Another research participant summed up her childhood as follows: “I had a really nice childhood, they tried to give us everything” (CODA). The parents’ approach is assessed by some of them as very active: “they always thought of something for the children, like a day at the traffic playground and bowling, we also went on different trips, hiking in the mountains, they enjoyed it a lot and so on, there were a lot of things” (CODA). For these individuals, their parents’ deafness did not significantly hinder their perception of a supportive upbringing. The absence of parent–child relationships is denied by some respondents in their cases as follows: “my Mom is my Mom, whatever she is, and my Dad is my Dad, whatever he is, if he doesn’t have a leg, a hand, he can’t see, he can’t hear, they are still my parents” (CODA), “me and my Mom, we are best friends, we talk about everything (...), and when I look at different families, they don’t have that, it’s just my Mum is my Mum and my Dad is my Dad and they’re together, I don’t know if it’s because they’re deaf, we’re closer to each other after all” (CODA).

One peculiar aspect of some of the positive evaluations is the tendency to equate their childhood experiences with those of more vulnerable families. For instance, one respondent stated: “there are children who had alcoholic parents, so they were much worse off” (CODA), “there are children who are from families of alcoholics or criminals and I think they are much worse off” (CODA), “when a person’s parents die it’s normal to take care of siblings” (CODA).

## Discussion

The following section presents interpretations of the themes described and the relationships between them. The primary subjects are parents, children, grandparents, and others. Additionally, Figure 1 illustrates the underlying factors causing the challenging situation for parents and their relatives.

Grandparents helping deaf parents is a common and understandable situation, as we can see in other research findings mentioned in the Background section. For a specific group of deaf parents of hearing children, however, the help takes a slightly different form. It often has a very specific goal beyond the usual forms of intergenerational support, namely, to introduce CODA to spoken language and to help with the demands of school activities that parents are not equipped to handle due to their disability. Grandparents thus relieve parents, especially in the areas of strain resulting from their disability. Additionally, they assist with matters that hearing parents may find unusual, such as providing assistance to the authorities and attending doctor’s appointments. In these instances, they remove the pressure of the communication barrier created by the lack of support from the authorities or as a result of the inadequate national education system. This grandparental approach is confirmed by Napier’s findings. She cites cases of grandparents interpreting and helping with the same situations as reported by our respondents (Napier, 2021). Grandparents thus lighten their part of the sandwich—not only by helping directly but also indirectly through helping their children, and their situation can thus be interpreted as a special form of inverted sandwich generation effect, as they help two generations (their children and grandchildren). Their demand for help may also be a consequence of the sense of dependence on others that their own childhood experiences with grandparents may have instilled in them. Consequently, grandparents may be the very subjects who created the dependency and simultaneously maintain it.

Furthermore, children’s involvement relieves parents of two sources of pressure—disability and lack of support, and focuses on interpreting and dealing with ordinary life situations. The role of closeness plays an important role here. Parents tend to rely on the already mentioned grandparents and children as first-choice solutions. However, when these are unavailable (or the often-mentioned rational choice not to use children for various difficult life situations), they turn to official help. This is not always available, however, and, for some CODAs, also lacking in credibility. These findings of our respondents are consistent with those of Plötner et al. who describe children’s involvement in helping others in an environment where they are alone and where there is someone else who could help. In their research, children apparently recognized that they alone were responsible to help; in other settings, there was a diffusion of their perceived responsibility (Plötner et al., 2015).

Children of deaf adults perceive the burden of childhood as challenging with the issues of early adolescence and independence emerging but are also viewed positively in some cases. These statements are supported by the research findings of Moroe and de Andrade (Moroe & De Andrade, 2018a), who address in particular the topic of preparedness for adulthood and independence, influenced by previous role reversal. Children of deaf adults are often faced with situations in which they must take responsibility for decisions that rely on them. This is due to the lack of support in childhood, which results in the absence of trusting relationships between children and parents. These individuals operate in different worlds.

In terms of helping within the neighborhood, a surprising group providing help to deaf parents was neighbors, all of whom were women. This relationship is based on the situation of deaf parents who make the most friendships among deaf people. These friends fail, however, to provide the necessary support to the parents in the areas of deficiency caused by the fact they are deaf or hard-of-hearing. Neighbors are thus the most physically proximate, especially when it comes to influencing children. Parents are also positive about the help of school, speech therapy facilities, and friends. This is a relatively common form of help, however, that also takes place with families of hearing parents.

The concept of parental dependency is regarded as a pivotal link between the mediated causes and effects, particularly in relation to the independence and isolation of CODA. As a consequence of the pressures associated with disability and a lack of institutional support, parents become dependent on others, including grandparents, children, and the environment. In some instances, this dependency can reach a point of helplessness, which in some cases is attributed to the upbringing of grandparents. The preceding themes demonstrate that the relationship between the generations is, to some extent, reversed.

Parental dependency is viewed as a key category linking mediated causes with effects (especially independence and isolation of CODA) with central subjects. As a result of the pressures of disability and lack of institutional support, parents become dependent on others (grandparents, children, and the environment) and, in some cases, reach the point of helplessness, which, for some of them, is to a certain extent caused by grandparents’ upbringing. Socially, the parents find themselves in situations characterized by dependence on other groups, which, in terms of the sandwich generation, helps them to relieve the pressure caused by disability and societal pressures. If we reverse Miller’s (1981) definition, we might describe the generation of parents as one in which the grandparents and children find that they are not quite independent and have reached a degree of dependence. A further reversion of the concept of the sandwich generation can be made with the definition provided by Bertini (2011, p. 1) in chapter 1.2. Some parents in our research show more of a need to be provided for by the outer parts of the sandwich (grandparents and children) and to have their needs met to an increased extent through this support. This relation is further augmented by the activities of surrounding generations. Grandparents provide assistance through brokering and by facilitating the introduction of children to the hearing community. The component of financial assistance that Grundy and Henretta (2006) include in the sandwich effect is not fulfilled here. Children contribute in a similar way through brokerage, which persists even when they no longer live with their parents. This completes the role reversal process described by Moroe and de Andrade (2018a) and aligns with the concept of reversing the pressure traditionally placed on the “sandwich generation” by broadening the space to support the generation of deaf parents in our research. This approach also redistributes the negative impacts, such as stress and mental strain, as described by Riley and Bowen (2016, p. 57) for the sandwich generation, away from the immediate sandwich generation and onto the peripheral generations of grandparents and children. These effects are evident in the statements of some CODA respondents, who reported a constant sense of pressure (“I don’t actually remember a time in my life when I had peace of mind”). This persists despite their adulthood and independence.

In some instances, however, there is a desire for independence, which is manifested by a refusal of assistance from children and grandparents and a description of their life as identical to that of their hearing parents. It is also noteworthy that while other research, for example, Listman and Kurz (2020), discusses the provision of support from the deaf community; this was not emphasized in the statements of the communication partners.

Children of deaf adults also experience a wide range of relationships—from independent parents to helpless parents and from parents with whom they have a warm relationship to parents with a distorted relationship—by the lack of affection or perceived lack of maturity of the parents. Children of deaf adults view such parents as their children. It is therefore apparent that the attachment of the central element of the sandwich to the surrounding parts can take many forms.

A contrasting element to the dependence of parents, and a partial consequence of their dependence, is the independence of children. The latter had to mature earlier due to the necessity of dealing with challenging life situations for which they were not sufficiently prepared. Independence was also associated with low support from parents in learning and coping with other school responsibilities, as well as CODA educational career decisions. As a consequence of the absence of profound emotional ties with their parents, CODAs in some instances departed from the family at an earlier age and sought compensation for these relationships elsewhere. This independence is then followed by a child–parent relationship that is based on practical assistance rather than an emotional connection.

The items in Figure 1 are complemented by assessments of parenting and childhood by communication partners. Both positive and negative evaluations of childhood and parenting appear in the statements. Some CODAs appear to be more negative, mainly due to feelings of lack of love and belonging from their parents. They evaluate their isolation negatively, caused by the influence of the isolation of deaf parents in general (e.g., on separate holidays without hearing children). There are also strong voices evaluating childhood neutrally and quite frequently—positively. In reflecting on their experiences, individuals with deaf parents have identified several benefits, including the development of empathy, patience, responsibility, and organizational skills. These findings are presented in a previous publication (citation will be added after the blind review). The integration of deaf parents into the deaf community has had a profound impact on their children’s lives, an observation that has been largely positively evaluated by communication partners. However, the interviews also highlight a unique challenge associated with bilingualism and biculturalism: The individuals do not fully identify with either culture. This phenomenon, termed “bilingual identity” by Preston (1996), describes how these individuals connect with the hearing community through spoken language, while their use of sign language to communicate with their parents and other deaf individuals integrates them into the deaf community. Despite this, neither culture is perceived as “their own.” This underscores the complex and multifaceted nature of their cultural and linguistic identities.

A crucial aspect that has been overlooked in numerous previous interpretations is the fact that the parents of the families under study are fulfilling their typical parental roles despite the shortcomings that have been mentioned. We must also consider the positive effects of caring for surrounding generations, which, in the theoretical context, we have associated with the concept of the sandwich generation. These positive outcomes are highlighted by Turgeman-Lupo et al. (2020) and Williams (2004), who link these benefits to the satisfaction derived from caring for both younger and older generations.

A particularly peculiar outcome of the interviews is the comparison of their childhood with fictional families of alcoholics, drug addicts, and so forth. These comparisons exhibit a similar pattern, prompting the question of whether this is an understanding of the world that is more firmly established and shared within the CODA group. Napier provides an intriguing context for this rejection of the stigma associated with underperforming families. One respondent (whose entire family was deaf) to her research reports that they were fortunate that no member of his family was hearing and so was not included in sympathetic statements about their parents and helping them. They thus avoided any negativity about the perception of his deaf parents (Napier, 2021). The phenomenon of perceiving the whole situation of family brokers and heritage signers in terms of stigma, sufficiency, and pride appears to be an intriguing topic worthy of further investigation.

Within this discussion and interpretation, we acknowledge the limitations of our investigation. A qualitative approach makes it difficult to capture the prevalence of this manifestation of relationships among communication partners. It also does not allow for the findings to be generalized to a larger sample of the population. Some CODA statements, reflecting events that are several decades old, may be problematic. This may be similar for parents, as the research inquiry is retrospectively oriented. A specific feature that may lead to a misunderstanding of the situation of parents and children is the particular design of the education and support system in the state in which the study was conducted. Deaf people there, in particular, had previously been subjected to severe isolation (residence in boarding schools from early childhood, prohibition of sign language use, segregation from mainstream schooling), which may have contributed significantly to the parental dependency we describe as the central category. This topic is discussed in greater detail in our earlier study, which will be made available for review upon completion of the blind review process. Additionally, further research could be conducted to examine grandparents’ perceptions of and relationships with parents as a central subject.

Another potential limitation is the transition between sign language and the language used for data analysis, interpretation, and dissemination. Despite our continuous efforts to ensure comprehension with communication partners and the use of an interpreter, we have been unable to fully delve into the nuances of meanings conveyed through the sign language interpreter and offer them to readers and listeners of our results. In this regard, we concur with Stone’s assertion that “(...) an original data (source language) is different from a translated utterance. Although some level of equivalence can be achieved, we cannot translate the source language in a way that is fully comprehensible to an audience who have not had contact with it” (Stone & West, 2012, p. 662).

One of the most significant sources of uncertainty regarding the interpretation of certain situations is the question of the extent to which parent–child relationships differ from those of the hearing population. As Singleton and Title conclude, deaf parents are “generally competent and caring, aware of their limited experience (…) and quite concerned about gaining access to (…) appropriate childrearing information” (Singleton & Tittle, 2000, p. 226). It is evident that certain elements are specific, yet it is unclear whether grandparents routinely assist parents with child-rearing duties, including school responsibilities. It is also uncertain whether parents, as adults, are expected to provide excessive care for their children. Additionally, it is unclear whether all children of hearing parents develop strong positive emotional relationships. This comparison and questioning are intended to acknowledge and soften certain strong statements that some readers might find problematic.

### Implications

The fundamental implications of the paper’s objective were to examine the functioning of the sandwich generation concept in relationships arranged around deaf parents of hearing children. If we accept the symbolism of the original sandwich concept, where its filling (the parents) is oppressed by the responsibilities attached to caring for dependent children and increasingly dependent parents, the situation appears to be inverted for the families under study. Grandparents tend to relieve their parents of some of their responsibilities by assisting with childcare and providing support to parents in navigating various authorities. However, there is a paucity of data describing the extent of responsibilities from grandparents to parents and vice versa. A similar phenomenon can be observed in the relationship between parents and CODAs. Children of deaf adults are subjected to considerable pressure from an early age to assist their parents. This pressure can take the form of direct requests from parents or indirect pressure from the environment, such as grandparents or various authorities. In some cases, there is even a role reversal in adulthood, whereby parents not only lose their duty of care but are placed in the role of children who are cared for. This situation can result in parents reaching the peak of their dependence. In this sandwich (if we can even say that it is still a sandwich), we observe a relaxation of the pressures on both sides. It is important to note, however, that this study is focused on identifying outliers and does little to confirm the normal functioning of families of deaf parents within which the pressures described in the literature may still operate. Thus, future research may wish to focus on the concept of the sandwich generation, specifically in relation to families of deaf parents and other groups similarly affected.

The practical implications of this issue primarily relate to the necessity of providing sufficient support to families of deaf parents, particularly in the form of barrier removal through the use of interpreters and a quality deaf education system. Children of deaf adults are a particularly vulnerable group in this respect. The absence of the mother tongue in the primary social group, coupled with the immediacy and spontaneity of helping parents with interpreting, creates specific conditions that affect the atmosphere of maturation and adolescence. In light of these considerations, it is imperative to implement effective strategies to assist this marginalized population.

## Footnotes

1 Some authors prefer the term people from deaf families (PDFs) “referring to heritage signers (...) who have grown up using sign language with deaf parents” (Napier, 2021, p. 31).
